# Fetal Heart Rate Patterns in Monochorionic Twins Following Acute Twin-Twin Transfusion

**DOI:** 10.1155/2009/498530

**Published:** 2009-04-06

**Authors:** Shunji Suzuki, Nao Iwasaki, Shuichi Ono, Miwa Igarashi, Tomoaki Murata

**Affiliations:** Department of Obstetrics and Gynecology, Japanese Red Cross Katsushika Maternity Hospital, 5-11-12 Tateishi, Katsushika-ku, Tokyo 124-0012, Japan

## Abstract

*Background*. We present here 2 cases of acute twin-twin transfusion occurred during vaginal labor in monochorionic-diamniotic twin pregnancies. *Case*. Fetal heart rate tracings showed tachycardia in the donor twin in the first case, while they showed reassuring patterns in both twins in the second case. *Conclusion*. These differences in changes of fetal heart rate in the donor twins following acute twin-twin transfusion may be resulted from the differences in amount of transfusion and elapsed time.

## 1. Introduction

Acute twin-twin transfusion is a problem of short duration and might result in a difference in hemoglobin concentration at birth between the donor and the recipient twins without the pathologic findings of chronic twin-twin transfusion syndrome such as oligohydramnios/polyhydramnios [1, 2]. Acute shifts of blood from one twin to the other twin are thought to be resulted from blood-pressure differences due to uterine contractions or changes in fetal positions [1–3]. We present here 2 cases of acute twin-twin transfusion occurred during vaginal labor in monochorionic-diamniotic twin pregnancies. Fetal heart rate tracings showed tachycardia in the donor twin in the first case, while they showed reassuring patterns in both twins in the second case.

## 2. Case 1

**Figure 1:**
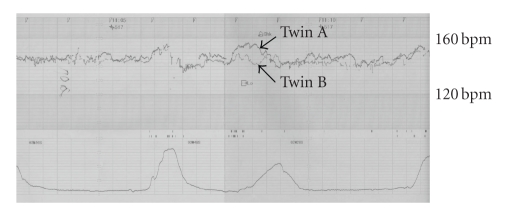
Fetal heart rate tracings during labor the latent phase of labor showing reassuring fetal heart rate patterns in both twins.

**Figure 2:**
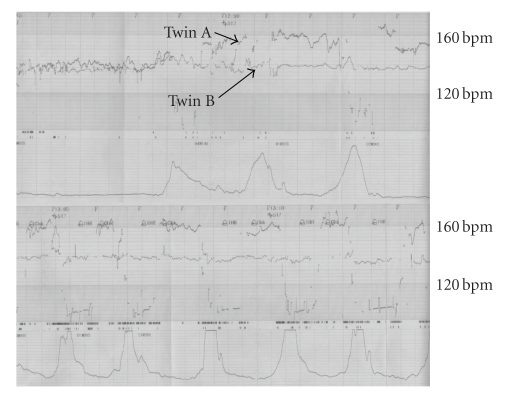
Fetal heart rate tracings during the active phase of labor showing the increased baseline of heart rate with variable decelerations in twin A.

A 26-year-old gravida 2 para 1 had labor induced by intravenous administration of oxytocin at 39 weeks' gestation. Her monochorionic-diamniotic twin pregnancy had progressed uneventfully based on weekly ultrasonic examinations. The amniotic fluid pockets and estimated fetal growth of both twins were normal at 5 days before the labor. On admission, her body temperature was 36.2°C and blood pressure was 124/78 mmHg. During the latent phase of labor, fetal heart rate tracings showed reassuring patterns in both twins. At this time, the base lines of twins A and B were 150 and 150 bpm, respectively, as shown in Figure 1. When the cervix was dilated 8 cm, the membranes of twin A were ruptured. Within 10–20 minutes, the fetal heart rate of twin A was increased to 170–180 bpm with repeated variable decelerations as shown in Figure 2. Thirty minutes later, a 3138 g male infant (twin A) with Apgar scores of 8 and 9 at 1 and 5 minutes, respectively, was born. His hemoglobin concentration was 11.4 g/dL (normal: 13–22 g/dL) with reticulocyte counts of 2.1% (normal: <7%). Six minutes after the delivery of the first twin, a 2720 g male infant (twin B), was delivered spontaneously with Apgar scores of 9 and 9 at 1 and 5 minutes, respectively. His hemoglobin concentration was 24.0 g/dL. He required intravenous infusion of 10% glucose for correction of polycythemia. The placenta was confirmed as monochorionic with 2 large superficial arterio-arterial anastomoses and 2 deep arterio-venous anastomoses. The growth and hemoglobin differences between the twins were 13% and 12.6 g/dL, respectively.

## 3. Case 2

A 37-year-old gravida 2 para 1 had labor induced by intravenous administration of oxytocin at 38 weeks' gestation. Her monochorionic-diamniotic twin pregnancy had progressed uneventfully based on weekly ultrasonic examinations. The amniotic fluid pockets and estimated fetal growth of both twins were normal at 4 days before the labor. On admission, her body temperature was 36.6°C and blood pressure was 136/80 mmHg. During the labor, fetal heart rate tracings showed reassuring patterns in both twins. The base lines of twins A and B were 140 and 145 bpm, respectively. A 2248 g female infant (twin A) with Apgar scores of 8 and 9 at 1 and 5 minutes, respectively, was born without difficulty. Her hemoglobin concentration was 11.2 g/dL with reticulocyte counts of 4.7%. Five minutes after the delivery of the first twin, the second twin, a 2078 g female infant (twin B), was delivered spontaneously with Apgar scores of 9 and 10 at 1 and 5 minutes, respectively. His hemoglobin concentration was 24.8 g/dL. She required intravenous infusion of 10% glucose for correction of polycythemia. The placenta was confirmed as monochorionic with 3 large superficial arterio-arterial anastomoses and 2 deep arterio-venous anastomoses. The growth and hemoglobin differences between the twins were 7.6% and 13.6 g/dL, respectively.

## 4. Comment

Although the multiple placental vascular anastomoses such as these 2 cases are nearly always present in monochorionic twin pregnancies, the incidence of acute twin-twin transfusion has been reported to be rare [1–6]. For example, we have encountered only these 2 cases of acute twin-twin transfusion (1.5%) in 132 monochorionic-diamniotic twin vaginal labors during the recent 5-year period.

Acute twin-twin transfusion is a diagnosis *per exclusionem* which has been thought to be mediated through large superficial low-resistance arterio-arterial or veno-venous anastomoses [1–4]. The possible criteria for acute twin-twin transfusion are: (a) difference in hemoglobin concentration at birth >5 g/dL between the twins, (b) the absence of pathologic findings of chronic twin-twin transfusion syndrome such as ologohydramniso/polyhydramnios, and (c) the absence of findings of twin anemia-polycythemia sequence such as high reticulocyte count in the donor twin [1–4]. The current 2 cases were suggested as having acute twin-twin transfusion based on their clinical findings, although it is not possible to prove these diagnoses absolutely conclusively.

In these cases, we observed 2 patterns of fetal hart rate tracings in the donor twins. One showed tachycardia, and the other showed normocardia. In some animals, there have been some observations in various fetal cardiovascular responses to fetal blood loss [7, 8]. Yoshihara et al. [7] observed that fetal heart rate did not change measurably during 40 ml per 2 hours of fetal hemorrhage in pregnant goats near term. Brace and Cheung [8] observed that the heart rate in fetal sheep was elevated by an average of 2 bpm at 3 to 5 hours after the fetal hemorrhage of 30% of their initial blood volume although it did not change measurably during the hemorrhage. These changes have been observed to be due to the changes in fetal plasma renin activity, arginine vasopressin, and norepinephrine following fetal blood loss [7, 8]. Therefore, the differences in changes of fetal heart rate in the donor twins of acute twin-twin transfusion may be resulted from the differences in amount of transfusion and elapsed time.

In the current 2 cases, there were no measurable changes in fetal heart rate tracings of the recipient twins following acute twin-twin transfusion. In some previous reports [1–4, 9], in addition, there have not been any significant comments about the fetal heart rate patterns in the recipient twins following acute twin-twin transfusion. Therefore, we expect the elucidation of heart rate patterns in the recipient twins by accumulation of the same case reports.
